# Buffalo as an ISM Center

**Published:** 1900-08

**Authors:** 


### BUFFALO AS AN ISM CENTER.

IT ONLY remains for some kindly light in the ocult sciences to open up a school for the dissemination of voodooism in this city, and then Buffalo will be amply supplied with all the isms and a full line of fake cures. Already there are in operation mills for the turning out of practitioners in christian science or Mothereddyism; osteopathy; chierotherapy, which by the way is a splendid opportunity for the never-do-wells in life; and now comes a disciple who advertises that he will teach weltmerism in a few lessons. This is an unexcelled chance. The advance agent of the new school, which by the way will cure a patient of any old disease in any old length of time for any old price, informs his prospective students that it is easy to make from $20 to $30 a day and not work nights either. Weltmerism, moreover, is a product of the wild and woolly west and makes a specialty of turning very ordinary and illy-educated mortals into full-fledged practitioners of the new cure by a hanky-panky, now-you-see-it-and-now-you-don't, sort of method. Incidentally, it cures the most grievous complaints and never goes near the patient. Distances makes no difference; in fact, that is the long suit of this school—the absent treatment scheme; but you must send your money in advance. If you don't get better it's the fault of conditions which surround you, not the fault of the weltmerist. If you haven't a disease send a dollar, and your business will increase before the dollar reaches the weltmerist. This statement is not an exaggeration; it is a solemn claim made in the organ of the cult. Just one more step in the line of progress and we shall read announcements offering unparallelled advantages in instruction in snake-skinning and bone-throwing by voodoos of long experience and great reputations.

